# The Doctor Will FaceTime You Now: Commentary on Telehealth in Cancer Care

**DOI:** 10.1093/oncolo/oyac044

**Published:** 2022-03-16

**Authors:** Jacqueline Feinberg, Yukio Sonoda

**Affiliations:** Gynecology Service, Department of Surgery, Memorial Sloan Kettering Cancer Center, New York, NY, USA; Gynecology Service, Department of Surgery, Memorial Sloan Kettering Cancer Center, New York, NY, USA; Department of Obstetrics and Gynecology, Weill Cornell Medical College, New York, NY, USA

## Abstract

The use of telehealth was already on the rise, but the COVID-19 pandemic caused an increase in the use of telehealth services. This commentary considers the benefits of and common concerns with the use of telemedicine in the oncology setting.

## Introduction

The COVID-19 global pandemic has shifted the adoption curve for telehealth within patient care. The use of telehealth, which already had been steadily rising over the past 10 years, began to rapidly accelerate with the advent of the pandemic starting in March 2020.^1^ The data are clear: not only is telehealth here to stay, but when appropriately and intentionally implemented and used, it can provide a wide range of benefits for our patients.

Broadly defined, telehealth includes synchronous communication via phone or video visits, asynchronous communication via messaging that may include photographs, and telemonitoring of treatment adherence or physiologic parameters. Multiple studies, including comprehensive systematic reviews, have demonstrated the efficacy, quality, and cost savings associated with telehealth care.^2-6^ As a result, the American Society of Clinical Oncology (ASCO) recently released guidelines on the integration of telehealth into routine oncologic care.^7^ However, patients and physicians have expressed some reservations and concerns with this method of care. Identified issues include the usability of software, the development of patient-doctor relationships, and the potential for missed findings without physical exams.^3,8^ As telehealth becomes even more ubiquitous, it is important to implement telehealth in a data-driven and common sense way to optimize patient outcomes, safety, cost, and patient and provider satisfaction.

## Impact of Telehealth on Patient Outcomes, Quality, Cost, and Access

Findings from an overview of 80 systematic reviews clearly demonstrated the benefits of telehealth in improving the quality of patient care, particularly with regard to chronic and severe illnesses.^5^ Across multiple disciplines, including congestive heart failure, diabetes, and other chronic conditions, studies have shown improved patient outcomes, decreased hospitalization, and improved medication compliance with telehealth. These data highlight the relevance of integrating telehealth within the current care constructs of oncologic treatment.

Patients and physicians have expressed high levels of satisfaction with telehealth.^6^ Furthermore, satisfaction ratings have been shown to increase alongside increased use of telehealth platforms, ie, the more patients use telehealth, the more they want to keep using it.^6,9^ In this month’s issue of *The Oncologist*, Quam and colleagues report that patients who already have established relationships with their physicians are more willing to incorporate telehealth into their care.^10^ Their findings highlight what we call the rule of common sense in telehealth implementation—behaviors that lead to patient satisfaction without telehealth are as critically important with the use of telehealth. In short, establishing a patient-physician relationship is key, regardless of the method of communication.

The findings of systematic reviews have also identified time and cost savings for patients, without increased time burden for physicians.^2,4,9^ In a cost-effectiveness analysis using data from more than 20 studies, 91% of telehealth interventions were found to increase quality of care and decrease costs.^4^ In another study, among patients with colorectal cancer enrolled in a new telehealth program, wait times for new appointments decreased, no-shows decreased, and patients felt they were more easily able to contact nurses.^5^ During a time in which financial toxicity is becoming more and more relevant to patients with cancer, interventions to reduce costs without negatively impacting quality of care are essential.^11,12^

Telehealth has also led to meaningful gains in access to care. Patients with limited travel means, who live in rural communities, or wish to include family during appointments can use telehealth to gain access to oncologists who would normally be out of their reach. For example, in rural Mexico, palliative care interventions using video chat, secure messaging, and phone calls were successfully implemented and used throughout the COVID pandemic.^3^ These types of interventions will likely be applied more widely going forward.

## Considering Common Concerns

Multiple studies have also identified concerns with telehealth,^8^ including patient apprehension with the lack of physical exams. In their well-conducted patient perspective study at a single institution in Minnesota, Quam and colleagues showed that 80% of patients were satisfied with their gynecologic cancer care telehealth services. Among patients with a preference for all in-person care, however, many indicated a level of concern about their physician “missing something” and believed a physical exam is “critical for detecting recurrence.”^10^ In another study, some patients reported concern that the “absence of an in-person visit harmed their treatment.”^8^

Given the potential benefits of telehealth, it is important to critically evaluate concerns about losing “necessary” in-person visits. In a retrospective review of various methods to detect ovarian cancer recurrence, Feinberg et al. showed that, in the context of a comprehensive screening program including routine tumor markers and imaging, physical exam findings were not the primary way recurrence was detected in any patient (of 147 recurrences) over 2 years.^13^ As such, it is clearly possible to implement telehealth for an appropriate subset of surveillance visits alongside routine tumor markers and imaging. Further studies to determine the additive value of in-person visits across multiple oncologic fields and treatment milestones will strengthen our field’s ability to select telehealth applications in a way that ensures outcomes are not compromised.

Other stakeholders, including physicians, have indicated concern about telehealth’s impact on the patient-physician relationship when breaking bad news and/or having complex conversations.^14^ Lastly, there are also technical concerns, such as adopting systems that are not easily or consistently usable, with technical road bumps and/or without sufficient support to guide patients and physicians if technical issues arise.^3,9,15^

## The Future of Telehealth in Oncology Care

Much of oncology care necessitates in-person treatment and management, often requiring complex patient-physician conversations and decision making, which makes it challenging to implement telehealth in this setting. To address some of these challenges, ASCO recently published guidelines^7^ that suggest telehealth can be appropriate for many types of visits, including on-treatment, result, survivorship, and palliative care visits. ASCO recommends in-person visits for initial visits, complex conversations, initiation of new therapy, and visits that require an in-person physical examination. Furthermore, to address concern regarding the impact on patient-physician relationship, ASCO highlights the importance of establishing the patient-physician relationship as a precursor to the use of telehealth and further notes that the use of telehealth for in-person visits should be based on patient preference.

Overall, we look forward to the ongoing impact of telehealth on the oncologic care provided to our patients. As healthcare providers, we must continue to shape the cancer care we provide, using evidence-based strategies to improve outcomes, quality, cost, and access. As the traditional models of communication and treatment of in-person care do not always translate to telehealth, and as the adoption of telehealth continues to rapidly gain traction, our duty is the continued innovation and implementation of new guidelines and best practices in the field. We encourage our peers to embrace the opportunities of telehealth and to help shape its optimal use.
